# Functional liposome loaded curcumin for the treatment of *Streptococcus mutans* biofilm

**DOI:** 10.3389/fchem.2023.1160521

**Published:** 2023-03-17

**Authors:** Zhimin Hu, Ying Tang, Bulin Jiang, Yue Xu, Siying Liu, Cui Huang

**Affiliations:** The State Key Laboratory Breeding Base of Basic Science of Stomatology (Hubei-MOST), Key Laboratory for Oral Biomedicine Ministry of Education, School and Hospital of Stomatology, Wuhan University, Wuhan, China

**Keywords:** liposome, curcumin, *Streptococcus mutans*, biofilm, deliver system

## Abstract

**Introduction:** Plaque biofilms, mainly formed by *Streptococcus mutans* (*S. mutans*), play an important role in the occurrence and development of dental caries. Antibiotic treatment is the traditional way to control plaque. However, problems such as poor drug penetration and antibiotic resistance have encouraged the search for alternative strategies. In this paper, we hope to avoid antibiotic resistance through the antibacterial effect of curcumin, a natural plant extract with photodynamic effects, on *S. mutans*. However, the clinical application of curcumin is limited due to its low water solubility, poor stability, high metabolic rate, fast clearance rate, and limited bioavailability. In recent years, liposomes have become a widely used drug carrier due to their numerous advantages, such as high drug loading efficiency, high stability in the biological environment, controlled release, biocompatibility, non-toxic, and biodegradability. So, we constructed a curcumin-loaded liposome (Cur@LP) to avoid the defect of curcumin.

**Methods:** Cur@LP functioned with NHS can adhere to the surface of the *S. mutans* biofilm by condensation reaction. Liposome (LP) and Cur@LP was characterized by transmission electron microscopy (TEM) and dynamic light scattering (DLS). The cytotoxicity of Cur@LP was evaluated by CCK-8 assay and LDH assay. The adhesion of Cur@LP to S. mutans biofilm was observed by confocal laser scanning microscope (CLSM). The antibiofilm efficiency of Cur@LP were evaluated by crystal violet staining, CLSM, and scanning electron microscope (SEM).

**Results:** The mean diameter of LP and Cur@LP were 206.67 ± 8.38 nm and 312 ± 18.78 nm respectively. The ζ-potential of LP and Cur@LP were ∼−19.3 mV and ∼−20.8 mV respectively. The encapsulation efficiency of Cur@LP was (42.61 ± 2.19) %, and curcumin was rapidly released up to ±21% at 2 h. Cur@LP has negligible cytotoxicity, and can effectively adhered to the *S. mutans* biofilm and inhibited its growth.

**Discussion:** Curcumin has been widely studied in many fields such as cancer, which can be attributed to its antioxidant and anti-inflammatory effects. At present, there are few studies on the delivery of curcumin to S. mutans biofilm. In this study, we verified the adhesion and antibiofilm of Cur@LP to *S. mutans* biofilm. This biofilm removal strategy has the potential to be translated into the clinic.

## 1 Introduction

Dental caries is one of the top three diseases in terms of the human incidence rate (Selwitz et al., 2007). One of the important pathogenic mechanisms of dental caries is the formation of a biofilm called dental plaque on the tooth surface (Valm, 2019). *S. mutans* is considered to be the main bacterium causing caries (Forssten et al., 2010). *S. mutans* can synthesize water-insoluble glucan using sucrose as the substrate through glucosyltransferase (Kawabata and Hamada, 1999). This process is conducive to the permanent colonization of *S. mutans* on the surface of tooth hard tissue and the production of extracellular polymeric substances (EPS) (Klein et al., 2015), and it also provides favorable conditions for the colonization of other bacteria and the maturation of biofilm (Kolenbrander et al., 2002). In addition, *S. mutans* has both acid production capability (Cui et al., 2019) and acid resistance (Baker et al., 2017). It can transport and metabolize various carbohydrates into organic acids, forming an environment rich in EPS and low pH, which is an important reason for the continuous destruction of tooth tissue (Lemos et al., 2019).

Currently, plaque control has been proven to be one of the important treatment measures to control dental caries (Addy, 1986). However, although commonly used antibacterial agents such as chlorhexidine can effectively inhibit biofilm and plaque accumulation (Brookes et al., 2020), there are many side effects, such as increased microleakage (Salama et al., 2015), tooth discoloration (Van Strydonck et al., 2012), and increased bacterial resistance (Kampf, 2016). Moreover, it has been reported that 0.12% chlorhexidine is more likely to form dental calculus on the tooth surface with dental plaque (Zanatta et al., 2010). The most serious side effect is severe allergic reactions to chlorhexidine mouthwash resulting in respiratory arrest and death (Pemberton and Gibson, 2012).

To avoid the numerous side effects of antimicrobials, researchers have turned their attention to other methods, such as cationic antimicrobial peptides (Lin et al., 2021; Sun et al., 2023), silver nanoparticles (Nafarrate-Valdez et al., 2022), and plant extracts derived from natural plants (Smullen et al., 2012). Our research group has been committed to the application research of plant extracts for a long time. Our previous research shows that many plant extracts, such as Epigallocatechin-3-gallate (EGCG) (Yu et al., 2021), quercetin (Yang et al., 2017), and resveratrol (Guo et al., 2021), have inhibitory effects on *S. mutans*. Curcumin is a yellow pigment in turmeric, which has been used as a dye and food flavoring since ancient times and has excellent biocompatibility (Gupta et al., 2013). Currently, curcumin has been shown to have antioxidant, anti-inflammatory, antibacterial, antifungal, antiviral, anticancer activities, and neuroprotective effects (Aggarwal et al., 2007). Curcumin is also a photosensitizer, so it can be used in photodynamic therapy (PDT) (Paschoal et al., 2015). In addition, it has been reported that curcumin may exhibit an effective anti-biofilm effect by regulating the expression of genes involved in bacterial quorum sensing (Kali et al., 2016).

However, low water solubility, poor stability, high metabolic rate, fast clearance rate, and limited bioavailability of curcumin have limited its clinical application (Yavarpour-Bali et al., 2019). Therefore, researchers have developed many methods to deliver curcumin, aiming to improve the utilization rate of curcumin *in vivo* (Ma et al., 2019). In recent years, liposomes (LPs) have become a widely used drug carrier due to their numerous advantages, such as high drug loading efficiency, high stability in the biological environment, controlled release, biocompatibility, non-toxicity, and biodegradability (Xing et al., 2016). Currently, the Cur@LP has been widely studied in treatment for cancer (Feng et al., 2017), Alzheimer’s disease (Mourtas et al., 2014), epilepsy (Agarwal et al., 2013), and acute myeloid leukemia (Sun et al., 2017). However, the study on curcumin targeting *S. mutans* biofilm in an oral environment is still rare.

**FIGURE 1:**
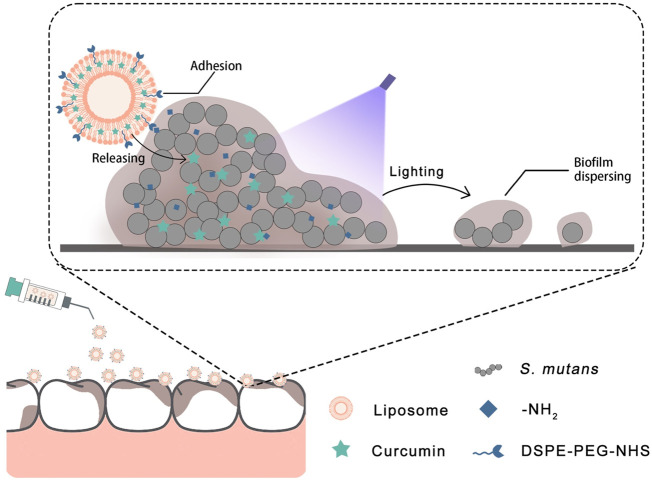
Antibiofilm principal diagram of the Cur@LP on *S. mutans* biofilm.

Based on the aforementioned details, we designed a liposome with adhesion properties to deliver curcumin into the biofilm. The surface of the liposome has an NHS group that can possess an activated carboxyl group to attach to the amino group (Wildling et al., 2011). In this strategy (Figure 1), the curcumin-loaded liposome can adhere to the surface of the biofilm and deliver curcumin into the biofilm more effectively. Then, the antibacterial photodynamic therapy was carried out by photoactivation of curcumin to achieve the effect of removing the *S. mutans* biofilm. The purpose of this study was to explore the removal efficiency of the Cur@LP on *S. mutans* biofilm. The results showed that the liposomes we designed effectively adhered to the surface of the *S. mutans* biofilm and achieved the corresponding antibacterial effect. In addition, *in vitro* experiments showed that the Cur@LP did not display significant cytotoxicity. We hope that this strategy can become a new approach to *S. mutans* biofilm removal treatment.

## 2 Materials and methods

### 2.1 Chemicals and reagents

DSPE-PEG-NHS [1,2-dioleoyl-sn-glycero-3-phosphoethanolamine-n-[poly (ethylenegly-col)]-hydroxy succinimide], L-α-phosphatidylcholine, curcumin [1,7-bis-(4-hydroxy-3-methoxyphenyl)-1,6-heptadiene-3,5-dione], and cholesterol were obtained from Aladdin Bio-Chem Technology (Shanghai, China). *S. mutans* Ingbritt and MC3T3-E1 osteoblast precursor cells were provided by the School of Stomatology, Wuhan University (China). Brain heart infusion (BHI) broth was purchased from Beijing Land Bridge Technology Co., Ltd. (China). Dimethyl sulfoxide (DMSO) was obtained from BioFroxx (Germany). Agar was obtained from Beijing Solarbio Technology Co., Ltd. (Beijing, China). The crystal violet staining solution and lactate dehydrogenase (LDH) cytotoxicity assay kit were purchased from Beyotime Biotechnology Co., Ltd. (Shanghai, China). Methanol, ethanol, dichloromethane, glutaraldehyde, and sucrose were obtained from Sinopharm Chemical Reagent Co., Ltd. (China). Cell counting kit-8 (CCK-8) was purchased from Dojindo Kagaku (Kumamoto, Japan). The α-modified essential medium (α-MEM) was obtained from HyClone (Logan, UT, United States). All reagents and chemicals were utilized as received.

### 2.2 Preparation of the curcumin solution

A solution of curcumin used as a photosensitizer was prepared and dissolved in dimethyl sulfoxide (DMSO) to obtain a stock solution of 20 mM. The stock solution was filtered by using a 0.22 μm pore size filter. On the day of the experiment, the stock solution was diluted in sterile phosphate-buffered saline (PBS) and stored at −20°C in the dark until use.

### 2.3 Preparation of the Cur@LP

L-α-phosphatidylcholine (1 mg), DSPE-PEG-NHS (0.4 mg), and cholesterol (0.25 mg) were dissolved in 10 mL of dichloromethane in a mass ratio of 20:8:5 in a 50 mL round-bottom flask. The solution was evaporated at 43°C by using a rotatory evaporator and the lipid membrane was gained. Then, 3 mL of PBS solution was added to the round-bottom flask with a lipid membrane. Once all of the lipid membrane is suspended in the solution, the suspension would be placed at 4°C overnight to efficiently hydrate the lipid membrane. Then, the liposome preparation was sonicated in a water bath for 1 h to remove all visible precipitates. Two polycarbonate membranes were placed in the LiposoFast™ extruder with 1 mL syringes (Avestin lnc., Ottawa, ON, Canada). The liposome solution was extruded by passing the suspension using 800, 400, and 200 nm polycarbonate membranes sequentially to obtain blank liposomes.

Similarly, apart from the addition of L-α-phosphatidylcholine (1 mg), DSPE-PEG-NHS (0.4 mg), and cholesterol (0.25 mg), a certain amount of curcumin was added to 10 mL of dichloromethane to achieve a curcumin concentration of 150 μM. The curcumin-loaded liposome (Cur@LP) was also prepared by the rotary evaporation method.

### 2.4 Characterization of the Cur@LP

The microstructure of the liposome was characterized by transmission electron microscopy (TEM, JEM-2100, Japan). A drop of the sample solution was placed on the copper grid with carbon film. After drying at room temperature, the samples were observed with TEM three times.

The mean diameters and zeta potential of the blank liposome and Cur@LP were obtained by dynamic light scattering (DLS, zeta sizer nano zs90, England).

To evaluate the dispersibility and stability of the LP and Cur@LP, we detected the particle size and polydispersity index (PDI) of the LP and Cur@LP at 1, 3, 5, and 7 days.

### 2.5 Encapsulation efficiency of the Cur@LP

The prepared Cur@LP was separated by centrifugation at 845 g for 15 min. The supernatant liquid was cracked by ethanol for 10 min. The concentration of curcumin was determined at a wavelength of 427 nm with an ultraviolet–visible spectrophotometer. The percent of encapsulation efficiency was calculated as follows:

$$Encapsulation\;efficiency\;\%=\frac{C_{2}}{C_{1}\times \frac{V_{1}}{V_{2}}}\times 100,$$

where C_1_ is the concentration of curcumin in dichloromethane, C_2_ is the concentration of curcumin in the supernatant liquid after centrifugation, V_1_ is the volume of dichloromethane, and V_2_ is the volume of the PBS solution added to the round-bottom flask with a lipid membrane.

### 2.6 *In vitro* release of the Cur@LP

After removing the nomadic curcumin, the dialysis bag containing the Cur@LP with a molecular weight cut-off (MWCO) of 15,000 was immersed at 37°C in a 30 mL mixture of PBS and methanol at a volume ratio of 3:2 and in an air shaker at 220 rpm. The release solution (3 mL) was taken out at a predetermined time, and an equal volume of a mixture of PBS and methanol was added. The percent of curcumin released from the Cur@LP was calculated as follows:

$$release\;\%=\frac{C_{t}\times 30+\sum_{i=1}^{i=n-1}C_{i}\times 3}{m}\times 100,$$

where C_t_ is the concentration of curcumin in the mixture at t time, n is the number of extractions of the release solutions, and m is the initial total amount of curcumin in the dialysis bag.

### 2.7 Culture of *S. mutans*

The frozen *S. mutans* was diluted in a fresh BHI medium (37 g/L) at a ratio of 1:100 in an Eppendorf tube or centrifuge tube and cultured at 37°C for 16–24 h. The concentration of *S. mutans* was 10^8^ colony-forming units (CFUs)/mL after culturing for 16–24 h. The concentration of *S. mutans* was determined at a wavelength of 600 nm with an ultraviolet–visible spectrophotometer. *S. mutans* biofilm was formed in dishes with the BHI (37 g/L) broth containing sucrose (17 g/L).

### 2.8 Detection of biofilm amount by crystal violet staining

*S. mutans* was diluted in a 48-well plate with the BHI broth containing sucrose at 10^5^ CFU/mL per well; curcumin of different concentrations was added and cultured for 24 h after exposure to blue light for 30 s (the irradiance of the blue light was 1,000 mW/cm^2^ with a final radiant exposure of 30 J/cm^2^). The antibacterial concentrations (mM) of curcumin without photoactivated effects were 0, 0.125, 0.25, 0.5, 1, 2, 4, and 8, respectively. The antibacterial concentrations (μM) of curcumin with photoactivated effects were 0, 0.125, 0.25, 0.5, 1, 2, 4, and 8, respectively.

After 24 h of co-culture, the supernatant was discarded and the bacterial biofilm was washed with the PBS solution three times. Then, 100 μL methanol was added and fixed for 10 min. After methanol removal, 100 μl of 1% crystal violet was added to the stain for 20 min. The wells were washed three times with the PBS solution after crystal violet removal. 100 μl ethanol solution was added to dissolve for 30 min. The samples were transferred to a new 96-well plate and determined at a wavelength of 630 nm.

### 2.9 Detection of cell viability by CCK-8 assay

MC3T3-E1 osteoblast precursor cells were cultured in alpha-modified minimum essential medium containing 10% (v/v) fetal bovine serum and 1% (v/v) penicillin/streptomycin in an incubator at 37°C and 5% CO_2_. The complete medium was replaced every 2 days, and the cells were digested by trypsin when the number of cells adhering to the wall reached 80%–90%. Then, the cells were seeded into 96-well plates at a density of 5,000 cells per well and incubated with 100 μl of the medium in each well for 24 h. Then, the liposome and different concentrations of curcumin or Cur@LP were added for 24 h, respectively. Each well was incubated with 100 μL of a mixture of the CCK-8 solution and complete medium at a volume ratio of 1:10 for 2 h away from light before measuring the absorbance at 450 nm by using an ultraviolet–visible spectrophotometer.

### 2.10 Detection of cytotoxicity by LDH assay

The cultivation of MC3T3-E1 osteoblast precursor cells was the same as 2.9. After the liposome, curcumin, or Cur@LP were added for 24 h, the 96-well plate was centrifuged by using a perforated plate centrifuge (Eppendorf) at 400 g for 5 min. Then, the supernatant was removed, and 150 μl of LDH release reagent from the LDH cytotoxicity assay kit was added to each well for 1 h. After centrifugation at 400 g for 5 min again, 120 μl of supernatant in each well was added in a new 96-well plate to mix with 60 μl of the LDH test working fluid at 25°C for 30 min away from light before measuring the absorbance at 450 nm by using an ultraviolet–visible spectrophotometer.

### 2.11 Antibacterial activity of the Cur@LP against planktonic *S. mutans*

*S. mutans* was cultured in the BHI broth. PBS, curcumin (Cur), liposome (LP), or Cur@LP was added to the *S. mutans* suspension diluted by 37 g/L BHI broth in Eppendorf tubes, respectively. The concentration of *S. mutans* in the Eppendorf tube was 10^5^ CFU/mL, while the concentrations of the curcumin and Cur@LP were 10 μM concurrently. Before 24 h of culture at 37°C, all Eppendorf tubes were irradiated for 30 s by blue light with a final radiant exposure of 30 J/cm^2^. Then, the concentration of *S. mutans* was determined at a wavelength of 600 nm with an ultraviolet–visible spectrophotometer.

### 2.12 Detection of Cur@LP adhesion to the *S. mutans* biofilm

*S. mutans* was seeded into confocal dishes with BHI broth containing sucrose to develop *S. mutans* biofilm. Curcumin or Cur@LP were added and co-cultured for 4 h. Then, the culture medium was removed, and after that, each dish was washed with PBS three times to remove unattached materials. *S. mutans* biofilms stained red with SYTO 59 and curcumin with green fluorescence adhesion on the biofilms were observed *via* confocal laser scanning microscopy (CLSM). Fluorescence images were analyzed by ImageJ. The experiment was repeated three times, and five photos were taken for every sample.

### 2.13 Detection of the antibiofilm activity of the Cur@LP

*S. mutans* was seeded into confocal dishes with the BHI broth containing sucrose to develop *S. mutans* biofilm, and then, PBS, Cur, LP, or Cur@LP was added, respectively. After culturing for 4 h, the culture medium was removed, each dish was washed with PBS three times, and then, new culture media were added to each dish. After 24 h, the supernatant was removed, each dish was washed with PBS three times, and the residual biofilms were stained red with SYTO 59 and observed *via* CLSM. Fluorescence images were analyzed by ImageJ. The experiment was repeated three times, and five photos were taken for every sample. The area of biofilm was calculated three times, and the data are presented as the mean ±SD.

### 2.14 Antibiofilm activity on *ex vivo* human teeth

The non-carious human teeth were obtained from clinical patients and cut into small cubes (5 × 5 × 2 mm, L×W×H). After sterilizing with ultraviolet irradiation for 2 h on both sides, respectively, the cubes were placed in a 24-well plate and immersed in the BHI broth with sucrose-containing *S. mutans* (10^5^ CFU/mL) with PBS, Cur, LP, or Cur@LP and incubated at 37°C for 4 h. Then, the culture medium was removed and each well containing cubes was washed with PBS three times, and new culture mediums were added in each well. Before 24 h of culture at 37°C, all cubes were irradiated for 30 s by blue light with a final radiant exposure of 30 J/cm^2^. Then, the culture medium was removed and the cubes were washed with PBS three times, and 2.5% glutaraldehyde was added to fix *S. mutans* biofilm on cubes for 24 h. After drying cubes with 30%, 50%, 75%, 90%, and 100% alcohol for 15 min successively, the cubes were used to take photographs of biofilms by using a scanning electron microscope (SEM).

### 2.15 Statistical analysis

All data are expressed as the mean ±SD under the condition of at least triplicate, and each experiment is repeated at least three times independently. The statistical analysis included one-way ANOVA and two-tailed unpaired Student’s t-test. We used GraphPad Prism 7.0 and Microsoft Excel 2019 software for data processing and statistical analysis (*p* < 0.05 means statistically significant).

## 3 Results

### 3.1 Characterization of the Cur@LP

**FIGURE 2:**
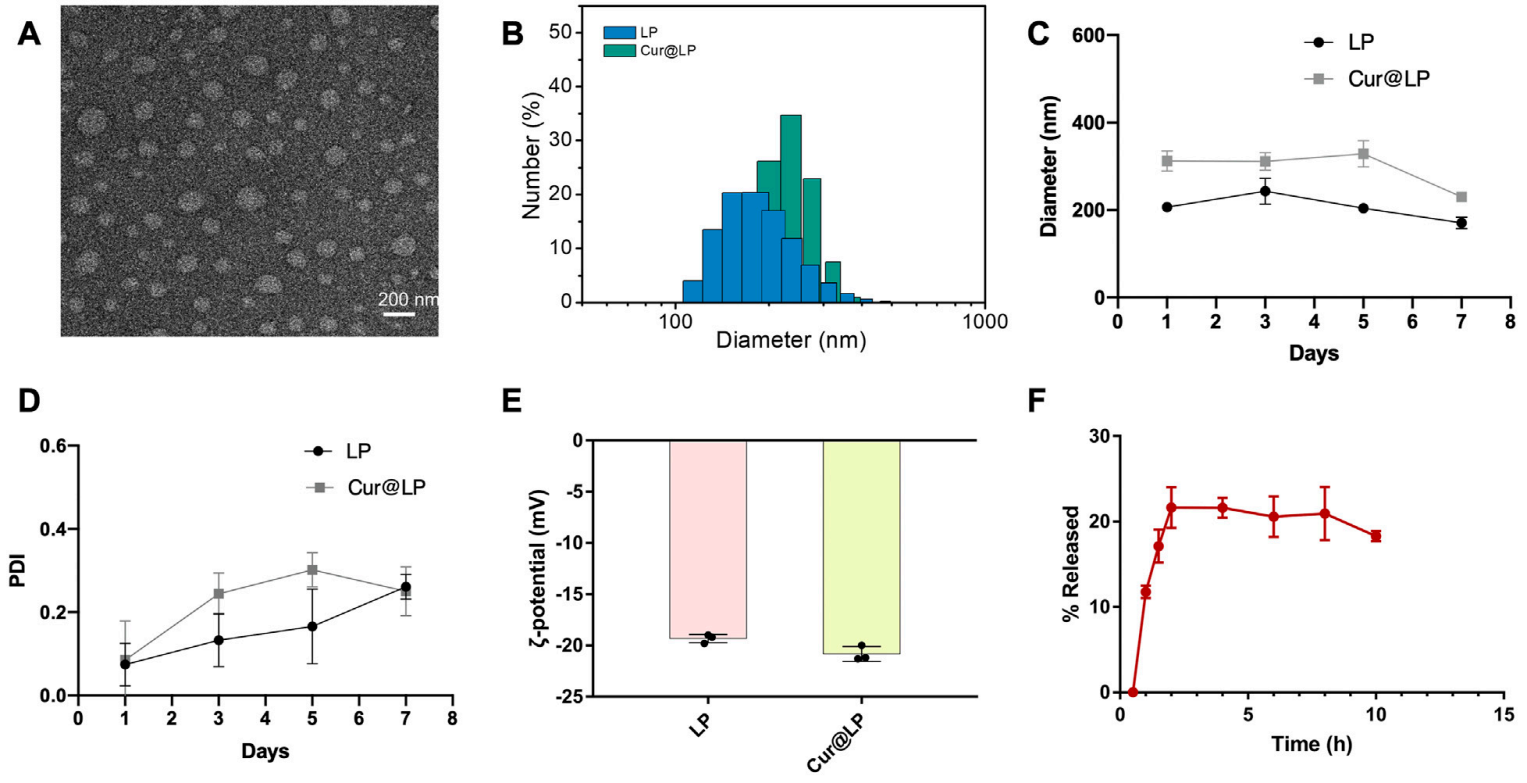
**(A)** Representative TEM micrograph of the Cur@LP. **(B,C)** Particle sizes of the LP and Cur@LP. **(D)** PDI of the LP and Cur@LP. **(E)** Zeta-potentials of the LP and Cur@LP. **(F)** *In vitro* release behavior of the Cur@LP at 37°C.

Figure 2A shows a TEM image of the synthesized Cur@LP, which formed a spherical vesicle-like morphology with good dispersion. According to the DLS results, the mean diameter of the liposome and Cur@LP was 206.67 ± 8.38 and 312 ± 18.78 nm, respectively, which are presented in Figure 2B. According to the results of the diameter and PDI of the LP and Cur@LP for a long time (Figures 2C, D), the diameter of the LP increased slightly at 3 days and then decreased slowly, while the diameter of the Cur@LP remained stable within 5 days but decreased at 7 days. The PDI of the LP and Cur@LP both showed a very low level at 1 day and then gradually increased over time. However, we found that the PDI of the Cur@LP decreased at 7 days, indicating the Cur@LP became more stable at 7 days. We speculate that curcumin released from the Cur@LP at 7 days is the possible reason why the diameter of the Cur@LP decreased at 7 days. In general, the PDI of the LP and Cur@LP both maintain a low level within 7 days. This result indicated that the Cur@LP has strong dispersion and stability at a certain time. Different particle sizes of liposomes and the Cur@LP proved that curcumin had been successfully loaded into liposomes. The ζ-potential of the liposome and Cur@LP, shown in Figure 2E, was ∼−19.3 and ∼−20.8 mV, respectively. The strong negative charge can maintain the stability of liposome suspension by charge repulsion.

### 3.2 Encapsulation efficiency and *in vitro* release behavior of the Cur@LP

The encapsulation efficiency of the Cur@LP was (42.61 ± 2.19) % by detecting absorbance at a wavelength of 427 nm. According to the results shown in Figure 2F, curcumin was rapidly released up to ∼21%, which was the highest value within 2 h. The total release remained constant for 2–8 h and decreased to ∼18.3% at 10 h.

### 3.3 Antibacterial effect of curcumin with photoactivation

**FIGURE 3:**
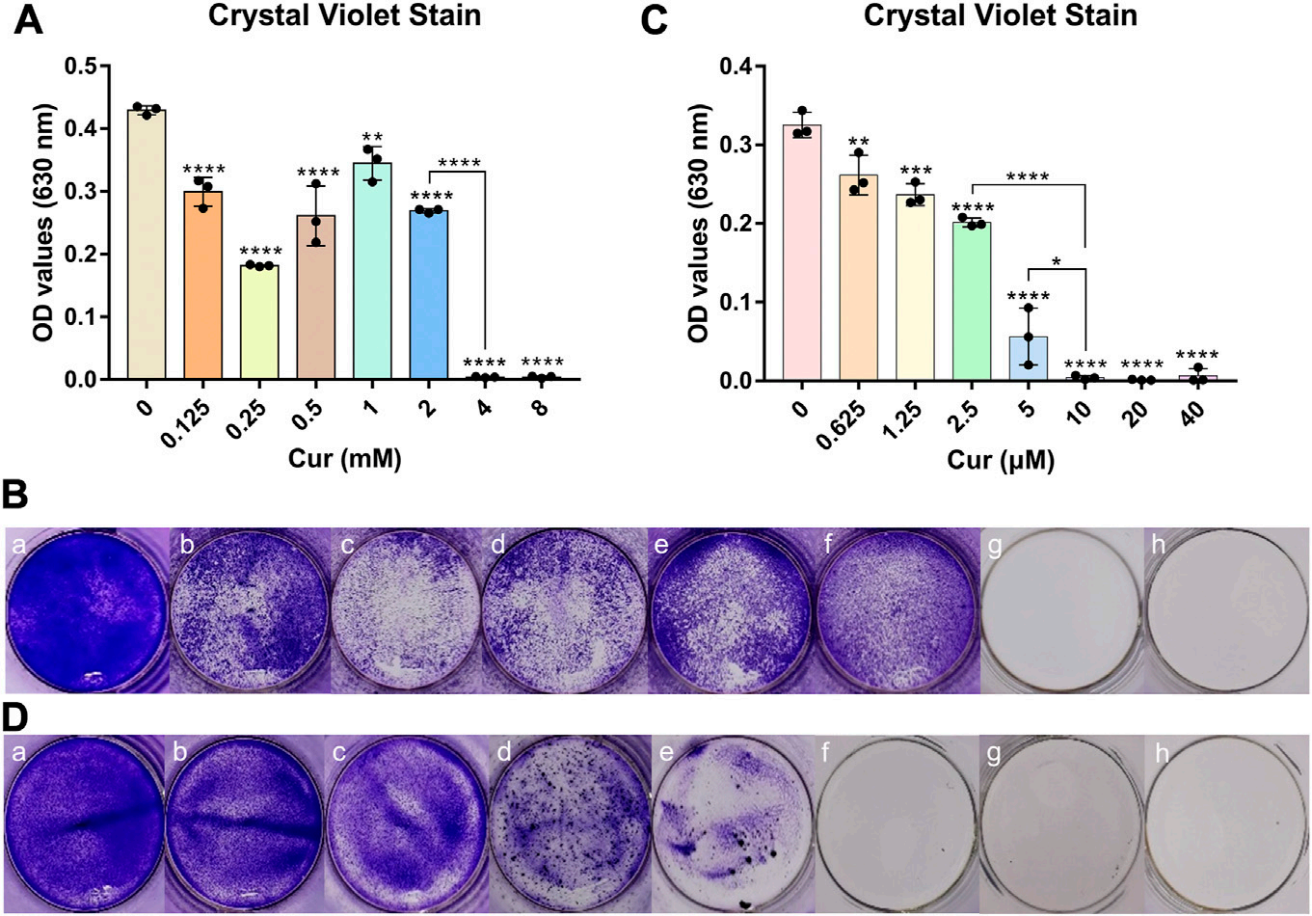
**(A)** Antibacterial properties of different concentrations of curcumin without photoactivation. **(B)** Crystal violet staining images of curcumins without photoactivation effects of Cur (mM) with (a) 0, (b) 0.125, (c) 0.25, (d) 0.5, (e) 1, (f) 2, (g) 4, and (h) 8. **(C)** Antibacterial property of different concentrations of curcumin with photoactivation. **(D)** Crystal violet staining images with photoactivation effects of Cur (μM) with (a) 0, (b) 0.625, (c) 1.25, (d) 2.5, (e) 5, (f) 10, (g) 20, and (h) 40. Statistical significance was calculated *via* two-tailed Student’s *t*-test. Data are presented as mean values ±SD.

As a photosensitizer, curcumin has an antibacterial effect even in the absence of photoactivation, which can be called the dark activity of agents. On the other hand, reactive oxygen species were produced by curcumin with blue light, which had the highest absorbance compared with other regions of visible light (Seidi Damyeh et al., 2020). Therefore, we determined the antibacterial activity of curcumin against *S. mutans* with photoactivation or not. According to the results shown in Figure 3, the minimum inhibitory concentration (MIC) for curcumin without photoactivation was identified at concentrations of 4 mM, while the MIC for curcumin with photoactivation was 10 μM. It is suggested that the antibacterial activity of curcumin with photoactivation was hundreds of times higher than that without photoactivation. Those results also emerged in staining images shown in Figures 3B, D. The MIC for curcumin with or without photoactivation is intuitively shown in Figures 3Bg, Df.

### 3.4 Detection of cell viability by CCK-8 assay

**FIGURE 4:**
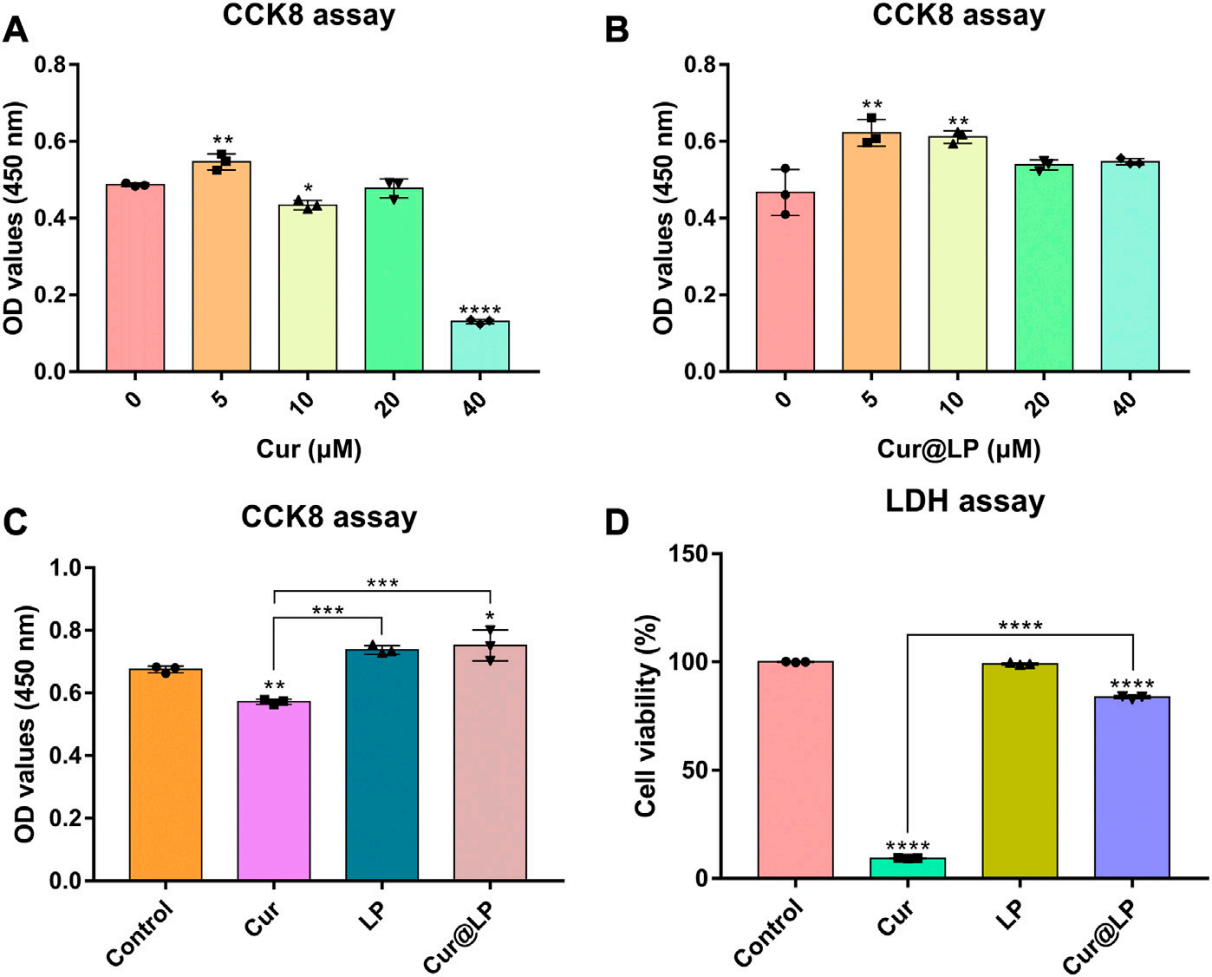
**(A)** Cell viability of MC3T3-E1 following cultivation with different concentrations of curcumin. **(B)** Cell viability of MC3T3-E1 following cultivation with different concentrations of Cur@LP. Cell viability of MC3T3-E1 co-cultured with PBS, Cur (10 μM), LP, or Cur@LP (10 μM) was detected by the CCK-8 assay **(C)** and LDH assay **(D)**. Statistical significance was calculated *via* two-tailed Student’s *t*-test. Data are presented as mean values ±SD.

The cell viability of curcumin and the Cur@LP was determined by CCK-8 assay. According to the result in Figure 4A, the viability of cells treated with curcumin at concentrations of 10 (*p* < 0.05) and 40 μM (*p* < 0.0001) all showed a significant decrease compared with the control group. However, the viability of cells treated with the Cur@LP at concentrations of 10 μM showed a significant increase compared with the control group (*p* < 0.01), and cells treated with the Cur@LP at concentrations of 40 μM showed no significant difference compared with the control group, respectively, shown in Figure 4B. Then, we compared the cell viability of curcumin, liposome, and Cur@LP at concentrations of 10 μM, and the same conclusion was obtained in Figure 4C. The curcumin group (10 μM) decreased significantly in viability compared with the control group (*p* < 0.01), while the Cur@LP group (10 μM) increased significantly (*p* < 0.05). ANOVA demonstrated a significant difference between the curcumin group and the Cur@LP group (*p* < 0.001). All the results of the cell viability assay proved that the cytotoxicity of curcumin was effectively reduced when it was loaded in the liposome.

### 3.5 Detection of cytotoxicity by LDH assay

The cytotoxicity of curcumin and the Cur@LP were determined by LDH assay. As shown in Figure 4D, though the curcumin group (10 μM) had obvious cytotoxicity, the Cur@LP group (10 μM) showed significantly decreased cytotoxicity compared with the curcumin group (10 μM). In addition, the LP group had no significant difference compared with the control group (*p* > 0.05).

### 3.6 Antibacterial activity of the Cur@LP against planktonic *S. mutans*

**FIGURE 5:**
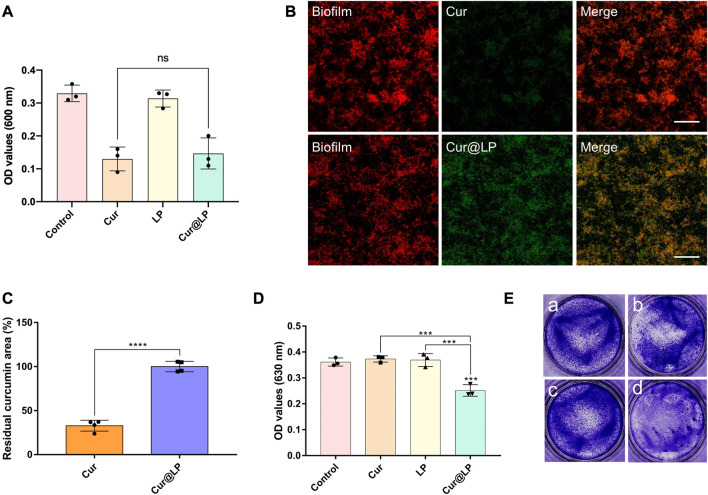
**(A)**OD value of the *S. mutans* planktonic culture after being treated with PBS, Cur, LP, or Cur@LP with photoactivation. The images **(B)** and quantification **(C)** of Cur or Cur@LP (green) adhesion on *S. mutans* biofilm (stain red with STYO 59). The OD value **(D)** and crystal violet staining images **(E)** of *S. mutans* biofilms after being treated with PBS, Cur, LP, or Cur@LP for 4 h. Statistical significance were calculated *via* two-tailed Student’s *t*-test. Data are presented as mean values ±SD.

To investigate the antibacterial activity of the Cur@LP against planktonic *S. mutans*, PBS, Cur, LP, or Cur@LP was added to the *S. mutans* planktonic culture. The concentration of *S. mutans* was determined at a wavelength of 600 nm with an ultraviolet–visible spectrophotometer. From the results shown in Figure 5A, we found blank LP had no significant effect on bacterial proliferation. The Cur and Cur@LP inhibited the growth of bacteria to a similar extent.

### 3.7 Adhesion activity of the Cur@LP

According to our design, the Cur@LP could attach to the surface of the biofilm to release curcumin into the biofilm efficiently. So, we detected the adhesion ability of the Cur@LP on the *S. mutans* biofilm by CLSM. As shown in Figure 5B, there was significantly more curcumin (green) adherent to the biofilm (stain red) in the Cur@LP group than in the Cur group. Figure 5C showed the quantitative result of the residual curcumin area between Cur and Cur@LP. The residual curcumin area of the Cur@LP group is significantly higher than that in the Cur group (*p* < 0.0001). These results indicated that LP can adhere to the biofilm surface in a relatively short time and promoted curcumin delivery into the biofilm.

### 3.8 Antibiofilm activity of the Cur@LP

**FIGURE 6:**
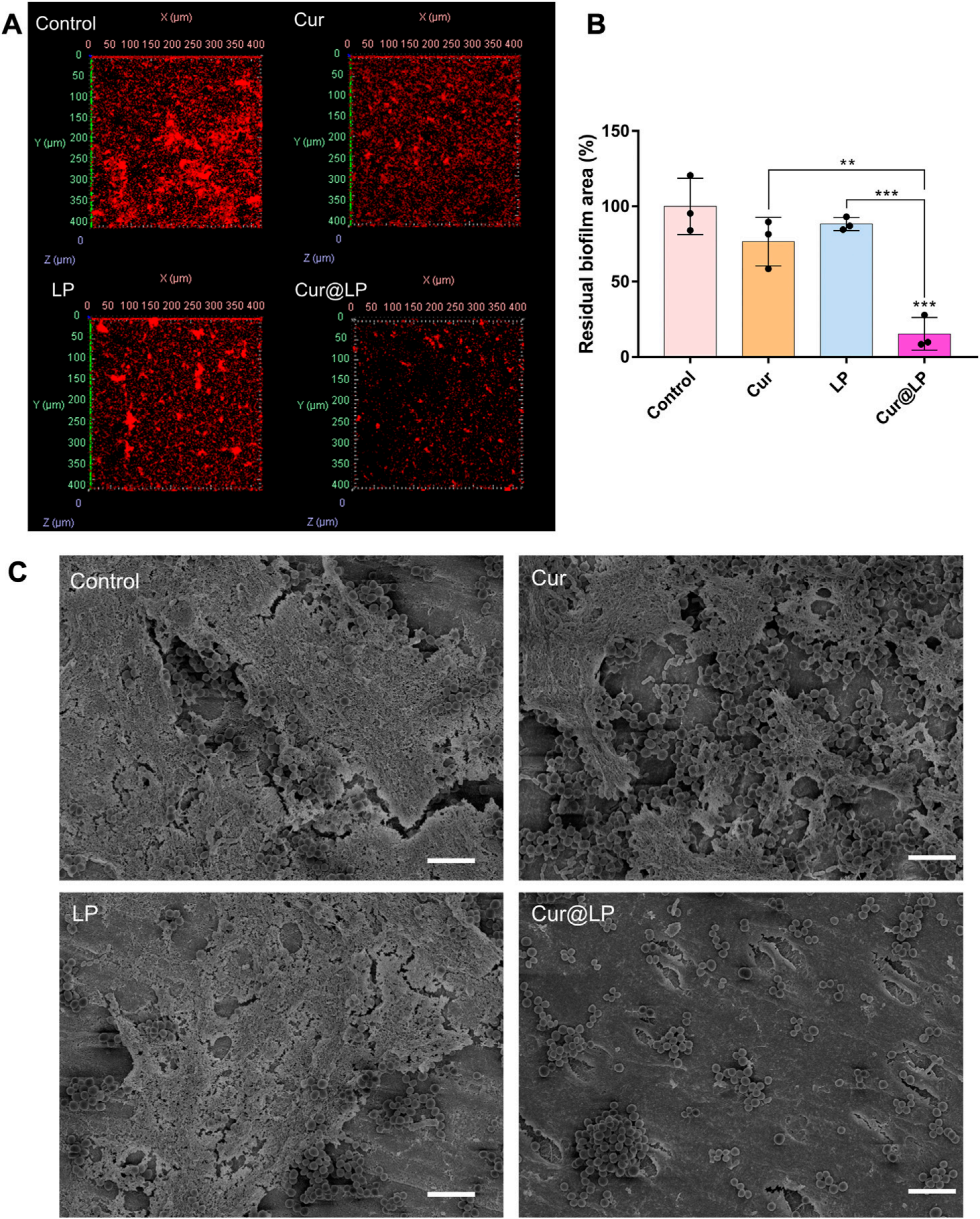
**(A)** Residual biofilm of *S. mutans* biofilm after treatment with PBS, Cur, LP, or Cur@LP for 4 h. The biofilms were stained red by STYO 59, and five photos were taken for every sample. **(B)** Statistical analysis of the biofilm removal area. Three biological replicates are shown. **(C)** Residual biofilm of *S. mutans* biofilm after treatment with PBS, Cur, LP, or Cur@LP on the human teeth. Statistical significance was calculated *via* two-tailed Student’s *t*-test. Data are presented as mean values ±SD.

After co-culturing of *S. mutans* and experimental groups for 4 h, the biofilm was preliminarily formed. Then, the old solution in the wells was taken out, and the new culture medium was added in all the wells for 24 h. The results shown in Figure 5D indicated that the biofilm formed in the Cur@LP group (10 μM) significantly decreased compared with other groups (*p* < 0.001). However, the biofilm in the Cur group (10 μM) showed no significant difference from that in the control group. This was probably due to the fact that free curcumin was washed at 4 h when the biofilm was preliminarily formed, while Cur@LP could attach on the biofilm and was not washed completely and released curcumin to play an antibacterial effect in the subsequent culture process. The staining images shown in Figure 5E also performed an intuitive display. In addition, the residual *S. mutans* biofilm after being treated with PBS, Cur, LP, or Cur@LP was observed by CLSM. As shown in Figures 6A, B, we found that the Cur@LP exhibited stronger biofilm removal efficiency than the other groups (*p* < 0.001).

### 3.9 Antibiofilm activity on *ex vivo* human teeth

Recently, many researchers have deeply studied the effects of various drugs on human dental biofilm (Liu et al., 2018; Wang et al., 2023). So, we decided to further perform anti-biofilm experiments on human isolated teeth. The results showed that the Cur@LP group had the least residual *S. mutans* biofilm when compared with other groups (Figure 6C), demonstrating that the Cur@LP possesses stronger anti-biofilm activity. We speculated that the possible reason is that curcumin alone cannot effectively adhere to the biofilm, and the use of liposomes loaded with curcumin greatly enhances its adhesion ability to better play the antibacterial effect.

## 4 Discussion

We constructed the liposome that can adhere to biofilm and is loaded with curcumin to exert the antibiofilm effect against *S. mutans*. Curcumin was loaded in the phospholipid bilayer, while the surface of the Cur@LP was incorporated by DSPE-PEG-NHS, an active derivative of PEG. The PEG skeleton has good hydrophilicity and lipophilicity, and the PEGylated phospholipid can significantly improve the stability of encapsulated drugs (Klibanov et al., 1990). For DSPE-PEG-NHS, the carboxyl group of NHS in the liposome can react with the amino group in the *S. mutans* biofilm to form the peptide bond, and then, the liposome can adhere to the biofilm (Pandey et al., 2018). After that, curcumin can be released from the liposome near the biofilm and exert an antibacterial effect by dispersing the biofilm under blue light irradiation, as shown in the diagrammatic sketch (Figure 1). Clinically, the blue light source has a wide range of applications that facilitates the application of Cur@LP solution or gel.

The synthesized liposome has a strong negative charge and can maintain the stability of liposome suspension by charge repulsion. The *in vitro* release data of curcumin suggested that curcumin was rapidly released within 2 h, reaching the highest value of ∼21%. However, the total release decreased to ∼18.3% at 10 h after maintaining the highest level at 2–8 h. Considering that the dialysate was a mixture of PBS and methanol with a volume ratio of 3:2, oscillating in an air shaker at 37°C and 220 rpm, we believed that curcumin became more unstable during the oscillation. Another possible explanation is that curcumin was partially decomposed under the photoactivated effect, even if light protection measures were taken throughout the whole process.

Curcumin, as a natural exogenous photosensitizer, collides with ground-state molecular oxygen and generates reactive oxygen species (ROS) under blue light excitation, also known as PDT (Luby et al., 2019). The ROS produced can oxidize the lipids in the cell membrane, leading to the disruption of membrane-binding proteins and resulting in apoptosis or necrosis of bacterial cells (Sperandio et al., 2013). In reality, although curcumin itself has a definite antibacterial effect, curcumin can exert a stronger bacteriostatic effect with photoactivation due to the role of PDT. This is also consistent with our experimental results that the antibacterial activity of curcumin with photoactivation was hundreds of times higher than that without photoactivation.

As a classical drug delivery carrier, the liposome has the greatest advantage of low cytotoxicity. According to the data shown in Figures 2C, D, the diameter of the Cur@LP remained at a stable level within 5 days, and the PDI of the Cur@LP increased gradually in a low range within 5 days. The results suggested that the Cur@LP has good dispersion and strong stability at a certain time. On the other side, we also noticed that both the diameter and PDI of the Cur@LP decreased at 7 days. This indicated that the Cur@LP showed better dispersion at 7 days. One possible reason we speculated is that curcumin was released from the Cur@LP at 7 days. The results of the cell proliferation assay suggested that curcumin showed obvious cytotoxicity with the increase in concentration. However, there was no significant cytotoxicity in the Cur@LP groups compared with the curcumin groups at the same concentration. Especially, the Cur@LP significantly performed cell proliferation compared with curcumin at a concentration of 40 μM. These results indicated that liposomes showed an obvious effect of protecting cells from the cytotoxicity of curcumin.

The result of the antibacterial activity of the Cur@LP against planktonic *S. mutans* showed that the Cur group and the Cur@LP group had no significant diffidence. This experimental result seems to prove that the Cur@LP has no obvious advantages over Cur against planktonic *S. mutans*. However, with the progress of subsequent antibiofilm experiments, we found that the greatest advantage of the Cur@LP we prepared is that it can adhere to the *S. mutans* biofilm when applied to the tooth. Therefore, we designed a set of experiments to observe the effects of different materials treated for a short period on biofilms. Each experimental group was co-cultured with *S. mutans* for 4 h to observe the fact that the liposomes could adhere to the preliminarily formed biofilm. Then, the old culture medium of all wells was taken out, and the new culture medium was added for 24 h. Curcumin has washed away since it could not adhere to the biofilm. On the contrary, most liposomes could retain and continue to release curcumin. Crystal violet staining results showed that the antibacterial property of the Cur@LP group was superior to that of the curcumin group (Figure 5A). Curcumin has a green fluorescence that can be observed by CLSM. As shown in Figure 6A, green fluorescence was significantly stronger in the Cur@LP group compared to the Cur group, possibly due to the fact that most of the curcumin in the Cur group was rinsed off, suggesting that the Cur@LP increased curcumin attachment. Similarly, the results of residual biofilm staining were also approximate to those of crystal violet staining. All the aforementioned results demonstrate that the designed liposome can adhere to the *S. mutans* biofilm and continuously release curcumin to exert antibacterial properties.

Curcumin has been widely studied in many fields, such as cancer (Maheshwari et al., 2006), which can be attributed to its antioxidant and anti-inflammatory effects (Hewlings and Kalman, 2017). In this study, we verified the application of curcumin in the oral field. Clinically, Cur@LP adherence to biofilm may be reduced due to severe mechanical movements such as tooth brushing. This will be the limitation of its application and the direction of future development.

## 5 Conclusion

In this study, a liposome with adhesion properties was designed and prepared. The liposome can adhere to the *S. mutans* biofilm and continuously release curcumin to achieve the antibacterial effect. As a natural photosensitizer, curcumin can be stimulated by blue light to play a stronger antibacterial effect. Clinically, the convenience brought by the widespread application of the blue light source and the adhesion ability to *S. mutans* biofilm will make the Cur@LP a broad application prospect.

## Data Availability

The original contributions presented in the study are included in the article/Supplementary Material; further inquiries can be directed to the corresponding author.

## References

[B1] Addy M. (1986). Plaque control as a scientific basis for the prevention of dental-caries. J. R. Soc. Med. 79 (14), 6–10. PMC1290094 3543355

[B2] Agarwal N. B. Jain S. Nagpal D. Agarwal N. K. Mediratta P. K. Sharma K. K. (2013). Liposomal formulation of curcumin attenuates seizures in different experimental models of epilepsy in mice. Fundam. Clin. Pharmacol. 27 (2), 169–172. 10.1111/j.1472-8206.2011.01002.x 22044441

[B3] Aggarwal B. B. Sundaram C. Malani N. Ichikawa H. (2007). Curcumin: The Indian solid gold. Adv. Exp. Med. Biol. 595, 1–75. 10.1007/978-0-387-46401-5_1 17569205

[B4] Baker J. L. Faustoferri R. C. Quivey R. G. Jr. (2017). Acid-adaptive mechanisms of *Streptococcus mutans*-the more we know, the more we don't. Mol. Oral. Microbiol. 32 (2), 107–117. 10.1111/omi.12162 27115703 PMC5498100

[B5] Brookes Z. L. S. Bescos R. Belfield L. A. Ali K. Roberts A. (2020). Current uses of chlorhexidine for management of oral disease: A narrative review. J. Dent. 103, 103497. 10.1016/j.jdent.2020.103497 33075450 PMC7567658

[B6] Cui T. Luo W. Xu L. Yang B. Zhao W. Cang H. (2019). Progress of antimicrobial discovery against the major cariogenic pathogen *Streptococcus mutans* . Curr. Issues Mol. Biol. 32, 601–644. 10.21775/cimb.032.601 31166181

[B7] Feng T. Wei Y. Lee R. J. Zhao L. (2017). Liposomal curcumin and its application in cancer. Int. J. Nanomedicine. 12, 6027–6044. 10.2147/IJN.S132434 28860764 PMC5573051

[B8] Forssten S. D. Björklund M. Ouwehand A. C. (2010). *Streptococcus mutans*, caries and simulation models. Nutrients 2 (3), 290–298. 10.3390/nu2030290 22254021 PMC3257652

[B9] Guo R. Peng W. Yang H. Yao C. Yu J. Huang C. (2021). Evaluation of resveratrol-doped adhesive with advanced dentin bond durability. J. Dent. 114, 103817. 10.1016/j.jdent.2021.103817 34560226

[B10] Gupta S. C. Sung B. Kim J. H. Prasad S. Li S. Aggarwal B. B. (2013). Multitargeting by turmeric, the golden spice: From kitchen to clinic. Mol. Nutr. Food Res. 57 (9), 1510–1528. 10.1002/mnfr.201100741 22887802

[B11] Hewlings S. J. Kalman D. S. (2017). Curcumin: A review of its effects on human health. Foods 6 (10), 92. 10.3390/foods6100092 29065496 PMC5664031

[B12] Kali A. Bhuvaneshwar D. Charles P. M. Seetha K. S. (2016). Antibacterial synergy of curcumin with antibiotics against biofilm producing clinical bacterial isolates. J. Basic Clin. Pharm. 7 (3), 93–96. 10.4103/0976-0105.183265 27330262 PMC4910474

[B13] Kampf G. (2016). Acquired resistance to chlorhexidine - is it time to establish an 'antiseptic stewardship' initiative? J. Hosp. Infect. 94 (3), 213–227. 10.1016/j.jhin.2016.08.018 27671220

[B14] Kawabata S. Hamada S. (1999). Studying biofilm formation of *mutans streptococci* . Methods Enzymol. 310, 513–523. 10.1016/s0076-6879(99)10039-9 10547815

[B15] Klein M. I. Hwang G. Santos P. H. Campanella O. H. Koo H. (2015). *Streptococcus mutans*-derived extracellular matrix in cariogenic oral biofilms. Front. Cell Infect. Microbiol. 5, 10. 10.3389/fcimb.2015.00010 25763359 PMC4327733

[B16] Klibanov A. L. Maruyama K. Torchilin V. P. Huang L. (1990). Amphipathic polyethyleneglycols effectively prolong the circulation time of liposomes. FEBS Lett. 268 (1), 235–237. 10.1016/0014-5793(90)81016-h 2384160

[B17] Kolenbrander P. E. Andersen R. N. Blehert D. S. Egland P. G. Foster J. S. Palmer R. J. Jr. (2002). Communication among oral bacteria. Microbiol. Mol. Biol. Rev. 66 (3), 486–505. 10.1128/MMBR.66.3.486-505.2002 12209001 PMC120797

[B18] Lemos J. A. Palmer S. R. Zeng L. Wen Z. T. Kajfasz J. K. Freires I. A. (2019). The biology of *Streptococcus mutans* . Microbiol. Spectr. 7 (1), 51. 10.1128/microbiolspec.GPP3-0051-2018 PMC6615571 30657107

[B19] Lin B. Li R. Handley T. N. G. Wade J. D. Li W. O'Brien-Simpson N. M. (2021). Cationic antimicrobial peptides are leading the way to combat oropathogenic infections. ACS Infect. Dis. 7 (11), 2959–2970. 10.1021/acsinfecdis.1c00424 34587737

[B20] Liu Y. Ren Y. Li Y. Su L. Zhang Y. Huang F. (2018). Nanocarriers with conjugated antimicrobials to eradicate pathogenic biofilms evaluated in murine *in vivo* and human *ex vivo* infection models. Acta Biomater. 79, 331–343. 10.1016/j.actbio.2018.08.038 30172935

[B21] Luby B. M. Walsh C. D. Zheng G. (2019). Advanced photosensitizer activation strategies for smarter photodynamic therapy beacons. Angew. Chem. Int. Ed. Engl. 58 (9), 2558–2569. 10.1002/anie.201805246 29890024

[B22] Ma Z. Wang N. He H. Tang X. (2019). Pharmaceutical strategies of improving oral systemic bioavailability of curcumin for clinical application. J. Control. Release. 316, 359–380. 10.1016/j.jconrel.2019.10.053 31682912

[B23] Maheshwari R. K. Singh A. K. Gaddipati J. Srimal R. C. (2006). Multiple biological activities of curcumin: A short review. Life Sci. 78 (18), 2081–2087. 10.1016/j.lfs.2005.12.007 16413584

[B24] Mourtas S. Lazar A. N. Markoutsa E. Duyckaerts C. Antimisiaris S. G. (2014). Multifunctional nanoliposomes with curcumin-lipid derivative and brain targeting functionality with potential applications for Alzheimer disease. Eur. J. Med. Chem. 80, 175–183. 10.1016/j.ejmech.2014.04.050 24780594

[B25] Nafarrate-Valdez R. A. Martínez-Martínez R. E. Zaragoza-Contreras E. A. Áyala-Herrera J. L. Domínguez-Pérez R. A. Reyes-López S. Y. (2022). Anti-adherence and antimicrobial activities of silver nanoparticles against serotypes C and K of *Streptococcus mutans* on orthodontic appliances. Med. Kaunas. 58 (7), 877. 10.3390/medicina58070877 PMC9323808 35888596

[B26] Pandey N. Hakamivala A. Xu C. Hariharan P. Radionov B. Huang Z. (2018). Biodegradable nanoparticles enhanced adhesiveness of mussel-like hydrogels at tissue interface. Adv. Healthc. Mater. 7 (7), e1701069. 10.1002/adhm.201701069 29205950 PMC5902656

[B27] Paschoal M. A. Lin M. Santos-Pinto L. Duarte S. (2015). Photodynamic antimicrobial chemotherapy on *Streptococcus mutans* using curcumin and toluidine blue activated by a novel LED device. Lasers Med. Sci. 30 (2), 885–890. 10.1007/s10103-013-1492-1 24249357

[B28] Pemberton M. N. Gibson J. (2012). Chlorhexidine and hypersensitivity reactions in dentistry. Br. Dent. J. 213 (11), 547–550. 10.1038/sj.bdj.2012.1086 23222325

[B29] Salama F. Balto H. Al-Yahya F. Al-Mofareh S. (2015). The effect ofcavity disinfectants on microleakage of compositerestorations in primary teeth. Eur. J. Paediatr. Dent. 16 (4), 295–300. 26637253

[B30] Seidi Damyeh M. Mereddy R. Netzel M. E. Sultanbawa Y. (2020). An insight into curcumin-based photosensitization as a promising and green food preservation technology. Compr. Rev. Food Sci. Food Saf. 19 (4), 1727–1759. 10.1111/1541-4337.12583 33337095

[B31] Selwitz R. H. Ismail A. I. Pitts N. B. (2007). Dental caries. Lancet 369 (9555), 51–59. 10.1016/s0140-6736(07)60031-2 17208642

[B32] Smullen J. Finney M. Storey D. M. Foster H. A. (2012). Prevention of artificial dental plaque formation *in vitro* by plant extracts. J. Appl. Microbiol. 113 (4), 964–973. 10.1111/j.1365-2672.2012.05380.x 22747830

[B33] Sperandio F. F. Huang Y. Y. Hamblin M. R. (2013). Antimicrobial photodynamic therapy to kill Gram-negative bacteria. Recent Pat. antiinfect. Drug Discov. 8 (2), 108–120. 10.2174/1574891x113089990012 23550545 PMC3740068

[B34] Sun D. Zhou J. K. Zhao L. Zheng Z. Y. Li J. Pu W. (2017). Novel curcumin liposome modified with hyaluronan targeting CD44 plays an anti-leukemic role in acute myeloid leukemia *in vitro* and *in vivo* . ACS Appl. Mater. Interfaces. 9 (20), 16857–16868. 10.1021/acsami.7b02863 28489348

[B35] Sun Z. Ma L. Sun X. Sloan A. J. O'Brien-Simpson N. M. Li W. (2023). The overview of antimicrobial peptide-coated implants against oral bacterial infections. Aggregate 2023, e309. 10.1002/agt2.309

[B36] Valm A. M. (2019). The structure of dental plaque microbial communities in the transition from health to dental caries and periodontal disease. J. Mol. Biol. 431 (16), 2957–2969. 10.1016/j.jmb.2019.05.016 31103772 PMC6646062

[B37] Van Strydonck D. A. Slot D. E. Van der Velden U. Van der Weijden F. (2012). Effect of a chlorhexidine mouthrinse on plaque, gingival inflammation and staining in gingivitis patients: A systematic review. J. Clin. Periodontol. 39 (11), 1042–1055. 10.1111/j.1600-051X.2012.01883.x 22957711

[B38] Wang Y. Zhou J. Yuan L. Wu F. Xie L. Yan X. (2023). Neighboring carboxylic acid boosts peroxidase-like property of metal-phenolic nano-networks in eradicating *Streptococcus mutans* biofilms. Small 19 (3), e2206657. 10.1002/smll.202206657 36394193

[B39] Wildling L. Unterauer B. Zhu R. Rupprecht A. Haselgrübler T. Rankl C. (2011). Linking of sensor molecules with amino groups to amino-functionalized AFM tips. Bioconjug. Chem. 22 (6), 1239–1248. 10.1021/bc200099t 21542606 PMC3115690

[B40] Xing H. Hwang K. Lu Y. (2016). Recent developments of liposomes as nanocarriers for theranostic applications. Theranostics 6 (9), 1336–1352. 10.7150/thno.15464 27375783 PMC4924503

[B41] Yang H. Li K. Yan H. Liu S. Wang Y. Huang C. (2017). High-performance therapeutic quercetin-doped adhesive for adhesive-dentin interfaces. Sci. Rep. 7 (1), 8189. 10.1038/s41598-017-08633-3 28811592 PMC5558009

[B42] Yavarpour-Bali H. Ghasemi-Kasman M. Pirzadeh M. (2019). Curcumin-loaded nanoparticles: A novel therapeutic strategy in treatment of central nervous system disorders. Int. J. Nanomedicine. 14, 4449–4460. 10.2147/IJN.S208332 31417253 PMC6592058

[B43] Yu J. Zhang Z. Guo R. Peng W. Yang H. Huang C. (2021). Epigallocatechin-3-gallate/nanohydroxyapatite platform delivery approach to adhesive-dentin interface stability. Mater. Sci. Eng. C. Mater. Biol. Appl. 122, 111918. 10.1016/j.msec.2021.111918 33641911

[B44] Zanatta F. B. Antoniazzi R. P. Rosing C. K. (2010). Staining and calculus formation after 0.12% chlorhexidine rinses in plaque-free and plaque covered surfaces: A randomized trial. J. Appl. Oral. Sci. 18 (5), 515–521. 10.1590/s1678-77572010000500015 21085810 PMC4246385

